# Uncommon Complications of Cystoscopy: Presentations of Concurrent Perirenal Hematoma and Candida albicans Sepsis

**DOI:** 10.7759/cureus.46602

**Published:** 2023-10-06

**Authors:** Katerina Jou, Nelson Barrera, Francisco J Gallegos Koyner, Salomon Chamay, Alejandro Nieto, Mahmoud M Ali

**Affiliations:** 1 Department of Internal Medicine, St. Barnabas Hospital Health System, New York City, USA; 2 Department of Clinical Medicine, City University of New York (CUNY) School of Medicine, New York City, USA; 3 Department of Internal Medicine, John H. Stroger, Jr. Hospital of Cook County, Chicago, USA

**Keywords:** flexible ureteroscopy, recurrent nephrolithiasis, subcapsular hematoma, chronic kidney disease (ckd), cystoscopy, candida infections, perirenal hematoma

## Abstract

Subcapsular hematoma (SRH) or perirenal hematoma (PRH) can be seen after trauma, interventional radiological procedures, urological procedures, anticoagulant medications, coagulation disorders, infections, and spontaneously in some patients. Within the urological procedures, PRH can occur after percutaneous nephrolithotomy and extracorporeal shortwave lithotripsy but has only been reported a few times after cystoscopy/ureteroscopy. Here, we present the case of PRH as a complication from cystoscopy with retrograde pyelography in a patient with underlying chronic kidney disease (CKD) and an extensive surgical history for nephrolithiasis. In addition to this, our patient had a further complication of sepsis by *Candida albicans*, of which the source is proven to be urinary, and it appears that the fungemia was triggered during the procedure as well. The diagnosis was confirmed by abdominal computed tomography (CT), and PRH was proven to resolve with conservative management on repeat imaging months later.

## Introduction

Subcapsular or perirenal hematoma (PRH) is a known and serious complication associated with urological interventions, specifically, percutaneous nephrolithotomy (PCNL) and extracorporeal shortwave lithotripsy (SWL). They may also occur due to trauma, interventional radiological procedures, or spontaneously in patients who are anticoagulated. Rarely, PRH occurs secondary to ureterorenoscopy (URS). The most common complications of URS are hematuria, infection, ureteral stenosis or perforation, and mucosal damage [1].

The average incidence of PRH secondary to URS is estimated at 0.45% [1]. An accepted hypothesis for the development of PRH is trauma to the pelvicalyceal system from guidewire insertion or as a result of increased intrarenal pressures leading to forniceal rupture. Predicted risk factors for PRH include a thinner renal cortex, moderate to severe hydronephrosis preoperatively, larger stone size, and the presence of underlying hypertension and urinary tract infection. Procedural risk factors include high perfusion pressures and prolonged operative time [1,2].

The most common symptoms of PRH are fever and back pain, along with signs and symptoms of anemia. Ultrasound is the easiest diagnostic modality, and confirmation is obtained with computed tomography (CT) imaging. Treatment is generally conservative; however, open surgery, percutaneous drainage, or vascular embolization may be required depending on the degree of bleeding, hemodynamic status, underlying comorbidities, and the presence of infection [2].

We present a case of a 42-year-old male who underwent cystoscopy with retrograde pyelography, placement of double-J ureteral stents, and lithotripsy who subsequently developed a right perirenal hematoma.

## Case presentation

A 42-year-old Hispanic male presented to the emergency department with five days of left lower quadrant abdominal pain rated 6/10 with radiation to the left lower back. He also reported dysuria, malaise, and nausea. Medical history was significant for well-controlled type 2 diabetes mellitus; hyperlipidemia; hypertension; bilateral nephrolithiasis with a past surgical history of bilateral laser lithotripsy on 05/2022, 07/2022, and 11/2022; and multiple bilateral double-J ureteral stent placements, with the last one being placed 10 days prior to admission secondary to bilateral intrarenal calculi with hydronephrosis and worsening post-obstructive acute kidney injury. His medications included amlodipine 5 mg daily; semaglutide 8 mg/2 mL, 2 mg every week; tamsulosin 0.4 mg daily; and rosuvastatin 10 mg daily.

On presentation, his blood pressure was 146/90 mmHg, heart rate was 80 beats/minute, temperature was 97.1°F, oxygen saturation was 99% on room air, and body mass index (BMI) was 38 kg/m^2^. He was alert, awake, and oriented. Physical examination was pertinent for bilateral costovertebral angle tenderness, with increased severity in the left flank. Initial laboratory values (Table 1) showed leukocytosis of 14,600/uL with neutrophilia (86%), hemoglobin of 13.9 g/dL, and a normal platelet count of 450,000/uL. His metabolic panel noted a creatinine of 1.9 mg/dL (baseline: 1.5 mg/dL), blood urea nitrogen of 33 mg/dL, glomerular filtration rate (GFR) of 39 mL/minute/1.73m^2^, glucose of 98 mg/dL, and hemoglobin A1C (HbA1C) of 6.4%. Urinalysis showed red-colored urine, a pH of 6.0 units, positive leukocyte esterase, white blood cell count (WBC) of >182/high-power field (HPF), red blood cell count (RBC) of >182/HPF, many bacteria, and few white blood cell (WBC) clumps. Urine culture ultimately was positive for *Candida albicans* (>50,000 to <100,000 colony-forming units (CFU)/Ml). The remaining initial laboratory results were unremarkable.

**Table 1:** Patient laboratory values throughout the clinical course GFR: glomerular filtration rate, WBC: white blood cell, HPF: high-power field, RBC: red blood cell

Laboratory study	Day 1	Day 2 (8 hours post-cystoscopy)	Day 3	Day 4	Day 8 (discharge)	1-month follow-up	Reference value
Hemoglobin (g/dL)	13.9	11.8	9.1	9.7	10.3	11.6	11.2-15.7
Leukocytes (#/uL)	14,600	17,600 (neutrophilia) (96.3%)	10,900 (neutrophilia) (91,8%)	9,900	10,500	7,300	4-10 (×10^3^/uL)
Platelets (#/uL)	340,000	238,000	264,000	302,000	412,000	363,000	150-450 (×10^3^/uL)
Creatinine (mg/dL)	1.9	1.8	1.5	1.3	1.3	1.5	0.6-1.2
Blood urea nitrogen (mg/dL)	33	20	18	21	18	21	8-23
GFR (mL/minute/1.73m^2^)	39	42	>60	>60	>60	51	>60
Glucose (mg/dL)	98	203	121	127	121	127	70-100
Hemoglobin A1c (%)	6.4	N/A	N/A	N/A			5.7-6.4
Urinalysis	Color: red, pH: 6.0, (+) leukocyte esterase, WBC: >182/HPF, RBC: >182/HPF	Color: red, pH: 6.0, (+) leukocyte esterase, WBC: >182/HPF, RBC: >182/HPF	N/A	Color: cloudy, pH: 6.0, (+) leukocyte esterase, WBC: >182/HPF, RBC: >182/HPF	N/A	Color: red, pH: 6.0, (+) leukocyte esterase, WBC: >182/HPF, RBC: >182/HPF	Color: clear, pH: 4.5-8, (-) leukocyte esterase, WBC: 2-5/HPF, RBC: 1-4/HPF

**Figure 1:**
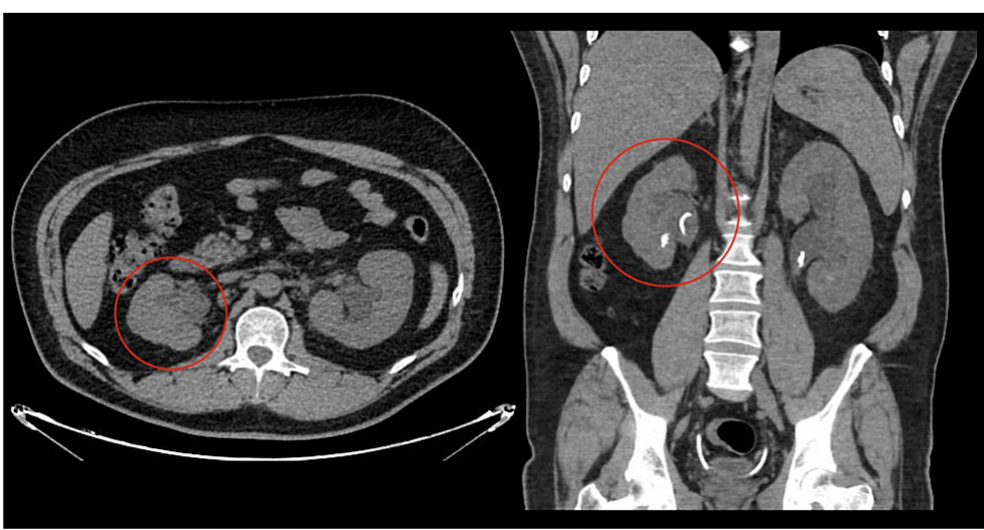
Axial and coronal views of the admission non-contrast CT The red circles show an atrophic right kidney with hydronephrosis and a double-J ureteral stent in place. A single non-obstructing stone is seen on the lower pole of the right kidney. CT: computed tomography

Computed tomography (CT) of the abdomen and pelvis (Figure 1) without contrast showed a new moderate left hydronephrosis with an obstructing left renal pelvic calculus measuring 1.1 cm. A left ureteral stent extending from the left renal pelvis distal to the calculus and with its distal tip in the bladder and a right ureteral stent from the right renal pelvis to the bladder were also reported. Additionally, several non-obstructing right renal calculi, with the largest measuring 0.5cm, were observed. The patient was diagnosed with obstructive nephrolithiasis complicated with pyelonephritis and was empirically started on IV ceftriaxone 1 g every 24 hours. The following day, the patient underwent cystoscopy with retrograde pyelography and placement of bilateral 6-French × 26 cm double-J ureteral stents. The placement of double-J stents was confirmed with fluoroscopy and cystoscopy. During the procedure, laser lithotripsy was unsuccessfully attempted in the left kidney and successfully performed over a stone in the right proximal ureter. The remaining stones in both kidneys appeared to be intraparenchymal.

Approximately eight hours after the procedure, the patient experienced severe right flank pain, quantified as 10/10 in intensity, with associated gross hematuria, chills, and nausea. His vital signs were as follows: blood pressure of 142/95 mmHg, heart rate of 122 beats/minute, temperature of 102.1°F, respiratory rate of 24 breaths/minute, and oxygen saturation of 96% on room air. Physical examination was remarkable for tenderness to palpation on the right flank. Given the high concern for sepsis, he received a bolus of 2 L of IV lactated ringers, 4 mg of IV morphine for pain, and 650 mg of oral acetaminophen. Blood work for sepsis workup was obtained, and antibiotics were changed from ceftriaxone to vancomycin 1,500 mg IV every eight hours and cefepime 1 gram IV every eight hours. A bedside abdominal X-ray showed bilateral double-J stents in the appropriate position. Laboratory studies showed an increase in leukocytosis to 17,600/uL and a drop in hemoglobin from 13.9 g/dL to 11.8 g/dL (Table 1). Coagulation studies were within normal ranges.

**Figure 2:**
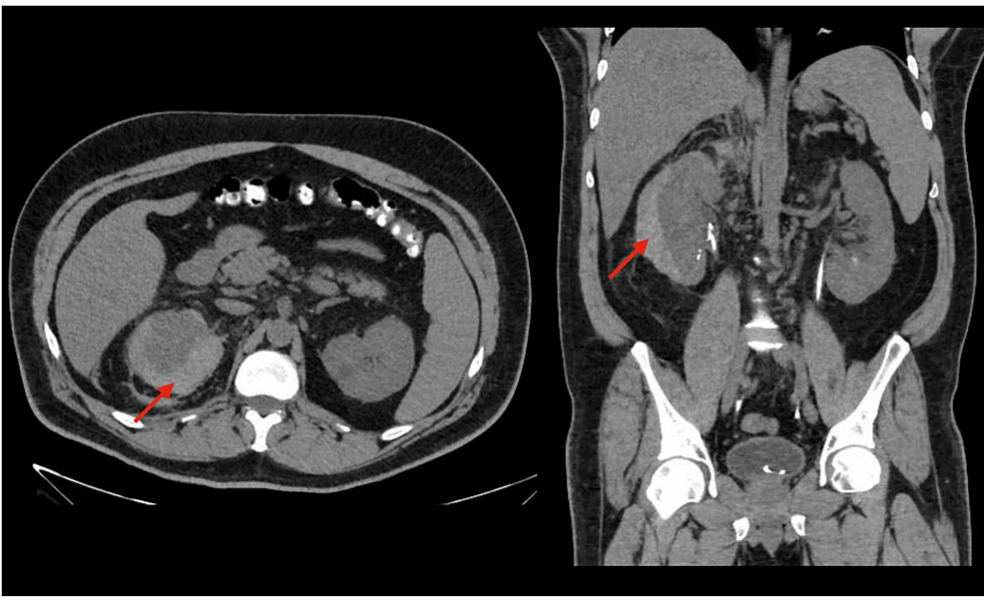
Axial and coronal views of a CT of the abdomen without contrast shown at the level of the kidneys The red arrows point toward a hyperdense lesion delimiting the renal capsule, measuring 50 HU, compatible with a perinephric hematoma. CT: computed tomography, HU: Hounsfield units

**Figure 3:**
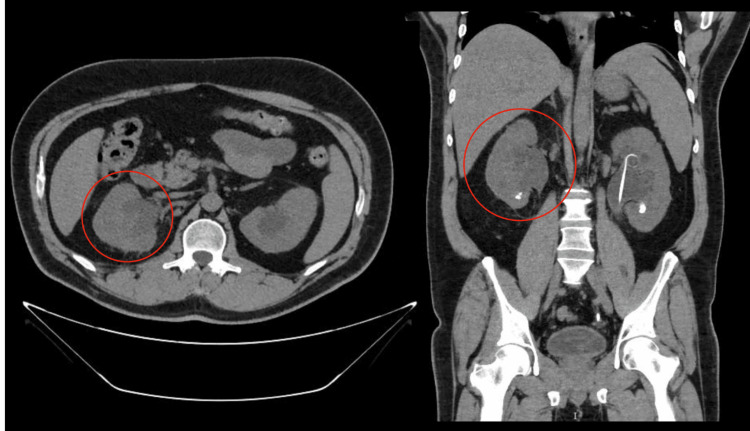
Axial and coronal views of a repeat CT of the abdomen without contrast done three months later of the episode showing near-complete resolution of the previously seen right perinephric hematoma (red circles), along with interval removal of the right double-J stent, and bilateral renal pelvic dilation, similar to prior examinations CT: computed tomography

The blood culture identification panel used in the anaerobic and aerobic samples was positive for *C. albicans*. Due to the presence of complicated *C. albicans* urosepsis, IV fluconazole 400 mg every 24 hours was started. Dilated fundus examination was negative for fungal ocular involvement, and transthoracic echocardiogram was unremarkable for valvular vegetations. Blood cultures after antifungal initiation remained negative. Despite the initial management, the patient was still experiencing right flank pain with gross hematuria and blood clots. Twenty-four hours after the procedure, hemoglobin further decreased to 9.7 mg/dL. At this time, the patient was normotensive but tachycardic. Due to the high suspicion of an ongoing retroperitoneal bleed, a CT of the abdomen and pelvis without contrast was obtained and revealed a well-defined subcapsular pocket of fluid collection measuring 7.2 × 4.0 × 2.3 cm over the right kidney, with a 50 Hounsfield units (HU) measurement compatible with a new perinephric hematoma (Figure 2). Additionally, hemorrhage was seen within the perirenal space and the lateral conal fascia, with normal positioning of bilateral double-J ureteral stents and non-obstructing bilateral renal stones. Because the patient was hemodynamically stable, a conservative treatment approach was pursued, and the right double-J stent was removed. Due to the self-resolution of hematuria within 24 hours and the stability of his hemoglobin, the patient did not require a blood transfusion. The patient was discharged on oral fluconazole 400 mg and completed six weeks of treatment. A repeat CT abdomen three months after cystoscopy showed near-complete resolution of the previously seen right perinephric hematoma, along with interval removal of the right double-J stent (Figure 3).

## Discussion

Subcapsular hematoma (SRH) or perirenal hematoma (PRH) is a rare complication of ureteroscopy and/or cystoscopy. Typically, it is identified as a complication of percutaneous nephrolithotomy, extracorporeal shock wave lithotripsy, or secondary to trauma and interventional radiological studies [1]. The reported incidence of PRH has varied widely in prior literature; Whitehurst and Somani (2017) cited an incidence of 0.15%-8.9%, with a propensity for developing in females (56.8%) and individuals over 50 years and with a mean stone size of 1.7 cm [1]. Others have recorded lower incidences with a narrower range of 0.15%-0.4% [2]. Risk factors for developing PRH include hydronephrosis, thin renal cortices, prolonged operative duration, preoperative urinary tract infections, larger stone size, ureteral stent usage, ureteral sheath usage, higher perfusion pressures, and prior renal operation/shock wave lithotripsy [1]. On the contrary, factors such as patient age, body mass index, diabetes mellitus, history of urolithiasis, hypertension, presence of multiple stones, and stone location have had questionable or statistically insignificant associations with the development of both PRH and SRH [3]. The patient not only presented with a prior history of ureteral stent placement but also had a history of hypertension, diabetes mellitus, bilateral hydronephrosis, nephrolithiasis, and laser lithotripsy, making him a prime candidate for developing PRH after ureteroscopy and cystoscopy. It is currently considered a grade IIb complication of the Satava classification system for intraoperative ureteroscopy complications [4].

With an initial patient presentation of fever and loin pain after ureteroscopy, PRH is then diagnosed with ultrasound and confirmed with CT imaging. What led to further evaluation of potential complications post-ureteroscopy was the presence of gross hematuria, unilateral right flank pain unresponsive to pain medication, associated tachycardia, and fever of 101.3°F. Additionally, Zhang et al. (2002) determined a sensitivity of 56% for diagnosing perirenal hematoma/hemorrhage by ultrasound and a sensitivity of 100% by CT imaging, with etiology identified with a sensitivity and specificity of 11% and 33%, respectively, by ultrasound, and 57% and 82%, respectively, by CT imaging [5]. Other disease pathologies that can be considered if ultrasound imaging was obtained include pyelonephritis with perinephric abscess, obstructive uropathy with perirenal urine leakage, and pseudoaneurysm; the wide array of potential disease processes suggested by ultrasound is what renders it less reliable for evaluating perinephric fluid collections [6].

The pathophysiology of PRH has not been clearly elucidated, but several etiologies have been hypothesized. Through ureteroscopy, trauma may be induced to the pelvicalyceal system with guidewire insertion, vigorous irrigation, ureteral access sheath placement, and double-J ureteral stent placement and consequently cause forniceal rupture, perinephric perforation, and PRH [1,2,7,8]. Bai et al. (2012) also hypothesized that increased intrarenal pressures and deformed/compressed renal blood vessels due to hydronephrosis that are subsequently relieved and recanalized may lead to vessel rupture and hematoma formation in the subcapsular space [2]. Furthermore, conditions such as urinary tract infections, hypertension, and chronic kidney disease can predispose the kidneys to bleeding in a state of inflammation, namely, anemia, thrombocytopenia, and platelet dysfunction [1].

The management and treatment of PRH secondary to ureteroscopy, cystoscopy, and/or other causes are not currently standardized but widely believed to be best through conservative means [1,4]. Several experts have provided recommendations on the treatment of PRH regardless of cause. The World Society of Emergency Surgery (WSES), the American Association for the Surgery of Trauma, and the European Association of Urology (EAU) recommend angioembolization for patients with large perinephric hematomas, but WSES-AAST limits angioembolization to require imaging demonstrating “active bleeding, increased bleeding risk, or in the setting of non-self-limiting gross hematuria” [9]. In our case, although there was a remarkable drop in hemoglobin (from 12 g/dL to 9 g/dL), hemodynamic stability was reached using crystalloids and hematuria and resolved eight hours after the procedure without the need for blood transfusion.

In a systematic review, Whitehurst and Somani (2017) found management strategies to primarily include blood transfusion and antibiotics (n=22, 55%) and percutaneous drainage (n=11, 27.5%), with the most extreme requiring surgical intervention with emergency angiography and open surgery in seven (17.5%) patients. However, percutaneous drainage placement may not be sought after due to a risk of infection [10]. Most PRHs will resolve spontaneously within six months, with a low degree of mortality; in one study, a patient died due to multiple organ failure after post-angiography nephrectomy [1].

Simultaneous with a presentation of perinephric hematoma post-cystoscopy was a blood culture positive for *Candida albicans*, which likely resulted from the cystoscopy, ureteral stent placement, and a plethora of other patient-related risk factors. Although candiduria is more understood, with a reported range of 6.5%-20%, concomitant candidemia from a urinary tract source appears to be a relatively rare complication (1%-8% of candidemia episodes) [11,12]. *Candida albicans* (35%-68%), *Candida glabrata* (8%-53%), and *Candida tropicalis* (3%-36%) are the most frequently isolated species found in urine for hospitalized patients [11]. Devices such as stents and catheters have been known to facilitate biofilm formation with *Candida* spp., and the degree of biofilm formation is associated with enhanced virulence [13]. Additionally, from a previously conducted retrospective study, researchers identified “underlying risk factors like urinary tract abnormalities (88%), use of antibiotics within 1 week (85%), underlying malignancies (73%), urinary tract obstruction (73%), and prior urological procedure within 2 weeks (73%)” may increase susceptibility to candidemia from a urinary source [12,14]. Our patient not only underwent urological procedures but also urinary tract obstruction with his initial diagnosis of nephrolithiasis and was empirically treated with ceftriaxone for potential urinary tract infection at admission as well. He was also a diabetic patient, despite having well-controlled blood glucose levels, which naturally predisposes to candiduria and thus candidemia [14].

More often, the timeline for developing candidemia after a urological procedure may not be immediately after “manipulation of the urinary tract” [13]. In their discussion, Vaidyanathan et al. (2013) cited that a possible explanation may be due to “(1) an ascending infection facilitated by urinary manipulation in the presence of candiduria; (2) subsequent development of micro- or macrofoci of renal parenchymal infection; and (3) limited or transient low-grade candidemia, perhaps precipitated by additional insults.” In their particular case, the researchers posited that the traumatic catheterization of the bladder and urethral mucosa permitted the dissemination of the fungus systemically, which may have occurred as well during our case [13].

Although most candidal colonization is asymptomatic and does not require treatment, the Infectious Diseases Society of America (IDSA) recommends that in non-neutropenic patients, echinocandins, such as caspofungin, are recommended as initial therapy (strong recommendation, high-quality evidence) with IV or oral fluconazole as an accepted alternative as initial therapy in patients “who are not critically ill and who are considered unlikely to have a fluconazole-resistant *Candida* species,” the latter of which was chosen as a treatment for our patient [14,15].

## Conclusions

Subcapsular hematoma (SRH) or perirenal hematoma (PRH) is a rare but potentially serious complication of ureteroscopy and/or cystoscopy. Its incidence varies widely in the literature, with several risk factors identified, such as hydronephrosis, prolonged operative duration, stone size, and prior renal operations. Prompt diagnosis is essential, and ultrasound and CT imaging are valuable tools for confirmation. The pathophysiology of PRH remains somewhat elusive, with trauma induced during ureteroscopy being one possible cause, along with increased intrarenal pressures and inflammation-related factors. Management of PRH is predominantly conservative, with blood transfusion and antibiotics commonly employed. Most PRHs resolve spontaneously over time, but some may require more intervention.

In the case discussed, the patient not only presented with PRH post-ureteroscopy but also exhibited candidemia, a relatively rare complication from a urinary source. The presence of risk factors such as urinary tract abnormalities, urinary tract obstruction, and prior urological procedures likely contributed to the development of candidemia. Ultimately, managing PRH and its associated complications requires a multidisciplinary approach, with close monitoring and appropriate interventions tailored to each patient’s unique clinical situation. Further research and standardization of treatment protocols will contribute to better outcomes for patients experiencing this challenging complication.
